# A Commercial Determinants of Health Perspective on the Food Environments of Public Hospitals for Children and Young People in High-Income Countries: We Need to Re-Prioritize Health

**DOI:** 10.3390/ijerph22040601

**Published:** 2025-04-11

**Authors:** Elena Neri, Claire Thompson, Caroline Heyes, Nancy Bostock, Wendy Wills

**Affiliations:** 1 Department of Nutrition and Public Health, University of Agder, 4630 Kristiansand, Norway; elena.neri@uia.no; 2 The Centre for Research in Public Health and Community Care (CRIPACC), University of Hertfordshire, Hatfield AL10 9AB, UK; w.j.wills@herts.ac.uk; 3 Cambridge University Hospitals NHS Foundation Trust, Cambridge CB2 0QQ, UK; c.heyes@nhs.net; 4 The Croft Child and Family Unit, Cambridgeshire and Peterborough NHS Foundation Trust, Cambridge CB21 5EE, UK; nancy.bostock@cpft.nhs.uk

**Keywords:** hospital food, healthcare settings, food environments, children, young people, diet, commercial determinants of health, critical narrative review

## Abstract

There is growing evidence that public hospitals in high-income countries—in particular, Anglo-Saxon neoliberal countries (USA, UK, Canada, New Zealand, and Australia)—have been engaging with food retailers to attract private capital and maximise their incomes in a drive to reduce costs. Added to which, public hospital food can have a substantial influence on the health of children and young people. However, there is still relatively little research on food for young people in healthcare settings. This is concerning, as an appropriate food intake is vital not only for the prevention of and recovery from diseases, but also for the physical growth and psychological development of young people. This critical narrative review examined the available evidence on hospital food provision, practices, and environments, as well as children’s experiences of hospitalization in high-income countries, drawing on both peer-reviewed articles and the grey literature. Our analytical lens for this review was the Commercial Determinants of Health (CDOH), a framework that necessitates a critical examination of commercial influences on individual, institutional, and policy practices relevant to health. Our findings illustrate the mechanisms through which the CDOH act as a barrier to healthy food and eating for children in hospitals in high-income countries. Firstly, hospital food environments can be characterised as obesogenic. Secondly, there is a lack of culturally inclusive and appropriate foods on offer in healthcare settings and an abundance of processed and convenience foods. Lastly, individualised eating is fostered in healthcare settings at the expense of commensal eating behaviours that tend to be associated with healthier eating. Public hospitals are increasingly facing commercial pressures. It is extremely important to resist these pressures and to protect patients, especially children and adolescents, from the marketing and selling of foods that have been proven to be addictive and harmful.

## 1. Introduction

Research from across disciplines and contexts has highlighted how food in healthcare settings, such as public hospitals (hereafter, ‘hospitals’), is often deemed as poor, which contributes to the perpetuation of poor health [1,2,3]. A hospital review carried out in the UK in 2020, for instance, found that 65% of patients stated that food impacted their overall experience of being in hospital in a negative way [4]. As inequalities continue to widen and pressures on healthcare systems increase, it is time to give due critical attention to food environments and food-related practices in healthcare settings, especially for children and young people. Although many studies have recognized the impact that public institutional food can have on children and young people, they have mostly focused on schools [5,6,7,8,9,10], while healthcare settings have been left out of the picture. This is concerning, as an appropriate food intake is vital not only for the prevention of and recovery from diseases, but also for the physical growth and psychological development of young people [11]. Rokach [12] reflects on how the hospital experience is particularly traumatic for children, who are much more vulnerable to the anxiety of understanding and adjusting to a new health condition and to the hospital environment. In this context, food is often the only element that can give a sense of relief, stability, and routine. Food, for instance, can be an important vehicle for children to express their emotions and feel in control when they are not able to do so verbally [13,14]. The few available studies on children’s experiences of hospitalization reveal the need for a more child-centred approach to food in healthcare [12,15,16].

Food served in hospitals has been heavily criticized, not only for its taste and quality, but also its nutritional properties, its lack of sustainability and cultural appropriateness, and even its safety [17]. Carter et al. [18] carried out a study in Canadian hospitals, finding that the organization of food provision and food quality were amongst the three major barriers to oral food intake for admitted children. Furthermore, since the early 2000s, researchers have bemoaned the fact that many hospitals in countries such as the US, New Zealand, Canada, Australia, and the UK have been cutting their expenditure in the public health sector and offering contracts to fast-food chains, convenience stores, and vending machines selling and marketing calorie-dense and ultra-processed foods high in salt, sugar, and saturated fat [19,20,21]. Statistics on childhood obesity in such countries are also alarming, ranging from 11% in the UK and Canada to 16% in Australia, 18% in New Zealand, and 20% in the USA [22].

The relationship that children and young people form with food during prolonged stays in hospitals is likely to follow them outside of the ward, having the potential to perpetrate, increase, or decrease social inequalities. A holistic overview of children’s hospital food environments and food-related practices in certain high-income countries—in particular, Anglo-Saxon neoliberal countries—is needed to inform recommendations for change and areas for further research.

This review aligns key factors that determine children and young people’s experiences of food in hospitals with the ways in which contemporary food systems entail an increasingly disconnected and mechanized relationship with food and eating [23], as well as with the penetration of powerful for-profit actors and their activities in public settings [24]. In short, we use the Commercial Determinants of Health (CODH) as a lens to critically synthesize the literature.

This approach is rooted in a critical stance against the industrial focus on profit and efficiency that has shifted society’s focus from food quality and food-related pleasure to profit and convenience [25]. In 2013, the WHO Director Margaret Chan [26] stated the following: “Efforts to prevent non-communicable diseases go against the business interests of powerful economic operators. In my view, this is one of the biggest challenges facing health promotion”. The same year, West and Marteau [27] introduced the term “commercial determinants of health”—which was then further refined by Kickbusch et al. [28] as a conceptual category to indicate “all the drivers and channels through which corporations propagate the non-communicable diseases pandemic”.

Maani et al. [29] have recognized how the CDOH are often understated, obscuring and deflecting attention from commercial sector responsibility for health inequalities and population harm. In the past few years, reviews have expanded CDOH definitions to incorporate the social structures and strategies that support the prioritization of corporate profit over population health and have called for specific research on how the CDOH operate in different settings [24,30,31,32,33,34]. Understanding and addressing the influence of corporate marketing practices is especially critical when it comes to vulnerable populations, such as children and young people [31]. Systems approaches that address the CDOH are needed to allow the development of healthier relationships with food and eating, improve population health, and reduce social inequalities [24].

This critical narrative review adds to the current literature by framing the evidence available on hospital food for children and young people in high-income countries (HICs) around the CDOH. This review focuses on Anglo-Saxon neoliberal countries—namely the USA, the UK, Canada, New Zealand, and Australia—where historical, political, and economic factors have facilitated the penetration of the CDOH into public settings [35]. Although such countries have many differences in their political and economic systems and ideologies, they are characterized by liberal market economies that provide freedom to private corporations and therefore the CDOH [36]. When it comes to public hospitals, such countries are also facing similar challenges, such as progressive privatization, decreases in public expenditure, widening social inequalities, and an increased incidence of non-communicable diseases.

Furthermore, we have focused on specific HICs in recognition of the fact that the CDOH manifest differently in such countries, especially compared to low- and middle-income countries (LMICs). In HICs, CODH research focus is typically on industries producing harmful commodities like processed foods, alcohol, and tobacco, and how these industries interact with regulatory frameworks and policy makers. For LMICs, CDOH research tends to centre on the disproportionate burden of diseases caused by unhealthy products and, in some cases, the way LMICs are targeted for both the export of products and labour exploitation [28,37]. Although it would be beneficial to explore and contrast the impacts of the CDOH on LMIC hospital food environments, this is beyond the scope of the current review. The aim of this paper was to explore the ways in which the CDOH shape hospital food environments and, thereby, impede healthy dietary intake for children in hospital in high-income countries.

## 2. Materials and Methods

A critical narrative review was undertaken to answer the following question: what are the mechanisms by which the CDOH act as a barrier to healthy food and eating for children in hospital in high-income countries? Rather than carrying out a systematic review, which focuses on a narrow question in a specific context, we opted for a narrative review, including a wide variety of studies, with the aim of providing an overall summary and of facilitating a critical, rather than inclusive or exhaustive, examination of the literature. We therefore did not aim for the inclusion of all relevant literature on the topic, but for thematic sufficiency [38]. The review was critical because we applied a theoretical lens (CDOH) to the analysis [39]. In this case, the review is underscored by the subjective and critical (but also evidence-based) assertion that the CDOH can be shown to negatively impact on hospital food provision and environments. Specifically, and in line with a systems approach to the CDOH, we sought to examine the literature with reference to different scales and levels through which (hospital) food environments operate [40].

The review was carried out between January and April 2022. Peer-reviewed articles were searched on Google Scholar using the search terms “hospital food”; “hospital food for children”; “hospital food environment”; and “children hospitalization”. Relevant articles were then selected based on their relevance to the conceptual lens. In order to collate an up-to-date picture of the current situation and guidelines regarding hospital food for children and young people, the literature was not limited to peer-reviewed articles but also included non-academic articles from newspapers with an international readership, national and international healthcare standards, official reports from private and non-profit organizations, and accounts by hospital food activists. Given that the term CDOH is relatively recent and emerged from turn-of-the century critiques of neoliberalism and marketisation, particularly in Anglo-Saxon neoliberal countries, only articles published since 2000 in such countries (USA, UK, Canada, New Zealand, and Australia) were selected as relevant. Article selection and interpretation were iterative. We stopped selecting articles once we had both established and sufficiently theorized the mechanism through which the CDOH shape children’s experience of food in hospitals.

After analysing the above material through a thematic approach, we grouped the issues connected to the CDOH affecting hospital food for children and young people into three thematic categories: hospital food environments; cultural inclusivity and appropriateness; and individual and social eating. These align with the aforementioned systems approach to CDOH [40], in which different scales and levels are examined. In this case, (i) built and institutional (hospital) food environments, (ii) provision and services (presence or absence of culturally inclusive food), and (iii) individual and group practices (eating behaviours that are fostered within these institutional and service contexts—as per i and ii) were considered.

## 3. Results

### 3.1. Obesogenic Hospital Food Environments

Many of the modern conditions and illnesses that lead children to hospitalization, such as tooth decay, type 2 diabetes, and other noncommunicable diseases, are mostly caused by the excessive consumption of certain foods, and may therefore be improved or even overcome through carefully planned, healthier diets [41,42]. The high burden of non-communicable diseases (NCDs), which are driven by the CDOH, is eminently preventable. The involvement of corporate actors in dietary public-health-related policy and practice further amplifies the challenges of addressing these burdens. Further, it keeps discussions of food and eating firmly in the arena of individual-level risk rather than systems and environments [40].

In line with this analysis, hospitals in Anglo-Saxon neoliberal countries seem to focus on curing symptoms rather than integrating food into the clinical treatment of patients and looking to more upstream solutions [43]. Several sources in the past few decades have exposed and criticized how hospitals in the UK and in the US have been serving children mass produced, branded food that is poor in nutritional quality and gustatory appeal, often heavily processed and high in salt, sugar, and saturated fat [44,45]. Despite the standards and guidelines aiming to improve the situation that have been designed and, at times, implemented ever since, they operate against the backdrop of provision, policy, and practice that is shaped, and even driven, by commercial considerations. Public–private partnerships in hospital logistics and procurement provide benefits for businesses at the expense of dietary public health. This results in poor food provision and unhealthy food environments [24].

Hospitals, as institutions, are pressured to increase efficiency, optimize space, and reduce costs. Consequently, they have been increasingly outsourcing meals for their inpatients from catering companies rather than cooking them on site [43]. Although this solution may reduce equipment and labour expenditure, it has many downsides: outsourcing contributes to the perception of food as a service that is separated, has lower priority than clinical treatment, and where cost savings can be achieved, shifting the focus from healing, quality, and pleasure to convenience and efficiency. Furthermore, outsourced meals are often made with ultra-processed ingredients, which have been linked to adverse health outcomes such as heart, kidney, and liver disease, cancer, and depression, especially if eaten from a young age [46,47]. Although there may be commercial benefits in outsourcing food provision, recent healthcare guidelines claim that these benefits do not outweigh the risks of implementing a culture where cooking from scratch is no longer prioritized and practiced, and therefore urge hospitals to bring back kitchens wherever possible [43,48,49].

Many hospitals have also been engaging with food retailers to attract private capital and maximize their income, offering in-hospital contracts to convenience stores, fast-food outlets, and vending solutions selling and promoting ultra-processed foods that are high in sugar, salt, and saturated fat to hospital staff and visitors. In 2000, an article regarding the opening of a McDonalds in the Starship Children’s Hospital in Auckland (New Zealand) exposed the general concern around the health effects of fast-food on children, but also around the way the public health system is increasingly prey to the predations of private enterprise, transforming patients—even at a young age—into consumers [19].

A study surveying the food services and health programs available in several Canadian and US children’s hospitals found that that most of them hosted fast-food franchise outlets or other outlets selling items of less nutritional value [such as convenience stores and soft drink vending machines], determining a suboptimal nutrition environment for patients and their families, as well as employees [20]. Sahud et al. [21] found that the presence of McDonald’s outlets within hospitals, beyond being associated with significantly increased purchase and consumption levels of McDonald’s food by outpatients, was also associated with the belief that the McDonald’s corporation supported the hospital financially, and with a higher perception of the healthiness of McDonald’s food itself. This final point is key in understanding the severity of the consequences of the CDOH in healthcare contexts, not only on patient health, but also in distorting their perceptions of for-profit brands and their aims.

The ‘obesogenicity’ of an environment has been defined as ‘the sum of influences that the surroundings, opportunities, or conditions of life have on promoting obesity in individuals or populations [50]. Obesogenic food environments comprise different elements, such as the availability and accessibility of unhealthy food, as well as food advertising and marketing, and are perceived to be a driving force behind the obesity epidemic and other noncommunicable diseases [50]. Apart from the abovementioned studies [19,21]—published at the beginning of the century, when tendencies to compromise between health and economic goals through franchising were just becoming prominent [19]—there is a lack of peer-reviewed literature examining hospitals as obesogenic food environments. Such research is needed, as many more hospitals have hosted fast-food chains, convenience stores, and vending machines selling and promoting calorie-dense foods and drinks ever since. Some hospital guidelines have already emphasized the moral and health implications of having certain kinds of convenience stores, fast-food outlets, and vending machines—some of the main components of obesogenic environments—inside public institutions that are aimed at practicing and promoting health and wellbeing, especially when it comes to children and young people [43,48,49,51]. Thinking about these food environments through a CDOH lens can be useful in bringing attention to how corporate profit is being prioritized, and how even hospitals are having to compromise between health and economic interests.

### 3.2. A Lack of Culturally Inclusive and Appropriate Provision

In high-income countries, a lack of access and knowledge around culturally appropriate foods often contributes to creating barriers to the inclusion and wellbeing of migrants and ethnic minorities [52,53]. Commercial pressures and the dominance of larger corporate providers can displace culturally appropriate, local, and minimally processed foods and retailers in favour of convenience, snack, and ultra-processed foods [54]. In this way, corporate food actors contribute to food swamps and deserts, resulting in dietary health inequalities [55]. Hospital food environments constitute no exception to this. Although the importance of respecting cultural diets in healthcare has been highlighted [56], hospitals’ food offerings still reflect the ethnocentrism of high-income societies and their markets. Raj [57] exposed how the lack of culturally inclusive food options in Western healthcare has harmful physical and psychological health effects on individuals from various ethnic minorities, such as Black, Asian, and Muslim communities. Although hospitals in some high-income countries have tried to improve their approach to multicultural patients through the “Migrant-friendly” hospital initiative, they still lack in care for them, especially from a paediatric perspective [58].

Different reviews giving recommendations for better hospital food [43,51,59] emphasise the urgency of designing menus that meet the needs of specific patient groups, such as Black, Asian, and other ethnic minorities and children. Culturally inclusive food options, rather than individualized and processed foods, must be made available. Being served culturally inappropriate food can be particularly problematic for children, who usually have high food neophobia (defined as the reluctance to eat non-familiar foods) and are less capable than adults of expressing their cultural dietary requirements to healthcare staff [60]. Their lack of power in this realm makes them vulnerable to imposing Western narratives around food and health. The standardised outsourced meals and Western fast-food outlets found in hospitals are unlikely to be able to provide culturally inclusive food options for children from diverse cultural backgrounds and ethnicities.

Paichadze et al. [31] state that the harms caused by the CDOH are amplified for vulnerable populations, such as children and young people or individuals from ethnic minorities. This is exacerbated for patients that belong to more than one vulnerable population at the same time, such as children from minoritised ethnic groups and different cultural backgrounds.

While the pervasive influence of the CDOH foster outsourced provision and individualized eating practices, there is a need to resist these trends and focus on inclusive and commensal meal options that have the added benefit of tending to be healthier [55]. There are currently very few highlighted and successful examples in this vein. The Memorial Sloan Kettering Cancer Centre (New York, NY, USA) improved physical and phycological patient recovery after prioritising the recreation of children’s favourite foods in its wards, while Ontario’s SicKKids hospital (Canada) replaced outsourced frozen meals with food cooked from scratch, prioritising children’s needs and preferences, as well as local procurement and catering staff ownership. Both cases happened under the lead of professional restaurant chefs and activists Pnina Peled and Joshna Maharaj, who were on a mission to improve hospital food and prioritised spending time with children and their families [61,62,63].

While these examples are inspiring and demonstrate the primary role of catering staff in the delivery of good, nutritious, safe, sustainable, and culturally inclusive food, they are single interventions that lack in a strategic, holistic, and reproducible approach. We also have no systematic measurement of the results that these changes achieved in the long term. Rather than leaving it to the discretion of individual food activists, who have limited tools and scope to make long-lasting change, governments should take responsibility and invest in designing evidence-based interventions and policies that can be robustly evaluated.

### 3.3. Individualised Eating Practices and a Lack of Social Eating

Kickbush et al. [28] explain how the individualization of food and eating is both a consequence and a facilitator of the “strategies and approaches used by the private sector to promote products and choices that are detrimental to health”. In the broader context, this has been fuelled by the shift towards contractualist, individualized neoliberal employment practices and the resulting changes in temporal patterns of work. In turn, this has desynchronized social lives and destabilized commensal eating practices, which are associated with healthier and less processed foods [64]. Fischler also recognized how individualistic approaches to eating—deemed as a consequence of the reorganization of modern industrial life—have resulted in Western societies eating increasingly alone rather than in groups [23].

These CDOH trends are replicated in healthcare settings, to the detriment of hospital food. Food and eating are typically approached at an individual patient level and focused on intake rather than physical and social environment [65]. While this is often necessary to accommodate dietary needs dictated by specific medical conditions and treatments, it arguably remains the norm even for patients who might benefit from opportunities for commensal mealtimes and experiences of hospitality (Ibid). Food is positioned as a commodity to be marketed and sold to individual consumers, rather than an occasion for learning and building relationships. Fischler [23] argued that this significant decline in commensality is associated with the rise in obesity, associated pathologies, and other problems involving public health.

The lack of research around commensality in hospitals suggests that healthcare institutions are rarely places where social eating is considered, practiced, or prioritised. In such settings, the individualization of eating characteristic of contemporary Western society is in fact exacerbated and amplified by impediments such as health conditions, dietary/intake mode requirements, staff and/or time restrictions, mobility impairments, and a lack of physical spaces and infrastructure. For children who are inpatients, this can have a severe and negative impact on their own as well as their families’ wellbeing. Coyne [15] found that most of the fear and anxieties hospitalized children expressed stemmed from the separation from friends, families, and familiar routines, of which social eating is a key component. Gelber [66] noted how parents should be more involved and allowed to feed their own children in hospitals, and that food should be provided to them as well as young patients so that they can eat together in the ward. The fact that commensality in the workplace has been linked to better staff cooperation, performance, and wellbeing [67] suggests that creating opportunities for hospital staff and young patients to eat together could potentially develop reciprocal understanding and relationships. Some hospitals have been setting up collective dining rooms in order to encourage social eating amongst older patients, which proved to be successful for improving their physical and psychological wellbeing, as well as increasing their food intake [68,69]. However, while there have been interventions to facilitate social eating in schools, there is no evidence of initiatives to enhance children and young people’s commensality being implemented within hospitals.

Social eating has been correlated to improve young people’s dietary quality and psychosocial wellbeing [70,71,72], as well as create a stronger sense of unity and cohesiveness amongst families [73]; it has been associated with enhanced mealtime experiences, social learning, and intrapersonal relationship development [74], as well as establishing children’s sense of normalcy, consistency, and routine [7]. Such evidence suggests that social eating should be explored as part of hospital treatment, especially for children and young patients. This means facilitating social eating amongst young patients, but also directing funds to providing food to parents, allowing them to eat with their children rather than having to leave the bedside to go and buy their own food from private businesses within hospitals [66].

## 4. Discussion

This review has looked at various aspects of food in hospitals through the lens of the Commercial Determinants of Health (CDOH), reflecting on how the commodification of food and eating in high-income countries has led to the interest of for-profit operators being prioritized over public health. The power of for-profit actors and their activities has penetrated public institutions, such as hospitals, which are meant to promote and protect health. This is particularly concerning when it comes to children and young people, who are more vulnerable to the CDOH. Many of the causes of children’s hospitalization, such as tooth decay, are in fact determined by the CDOH in the first place [29].

Hospitals are increasingly facing commercial pressures [28]. It is extremely important to resist these pressures and to protect patients, especially children and adolescents, from the marketing and selling of foods that have been proven to be addictive and harmful [75,76]. The commercial world harnesses Western neoliberal narratives that construct children as consumers, and this tendency is reflected in how food is dealt with in public spaces [13]. McDonald et al. [20] state that the adverse effect of unhealthy hospital food environments may be magnified because hospital staff, patients, and their families represent a relatively captive consumer market, while Washington [45] speaks of modern hospital patients as the “ultimate captive audience”. Institutions that are meant to protect children and young people—such as schools and hospitals—should be sites of resistance from the CDOH. Beyond being an ethical obligation, this is also likely to be better for the institutions themselves. While saving money on food and offering high-profit contracts to popular convenience stores and fast-food chains may seem a quick solution, it will in fact cause the healthcare system to incur much greater costs in terms of increased hospitalizations, slower recovery rates, and aggravated social inequalities in the long term.

A culture shift and system-wide change need to occur: food can no longer be seen as a leverage space to maximize efficiency and reduce costs, or as an opportunity for profit, but needs to be considered just as important as clinical treatment. As pinpointed by Smith [77], any genuine attempt to promote health must recognize the importance of the total social environment in which health behaviour is entrenched, rather than focusing on individual short-term solutions. As it stands, commercial interests have a profoundly negative impact on the dietary health of children in wider society. Children are targeted by corporate actors in the food system, especially in terms of marketing [78]. The downstream consequences of the CDOH are centred around the rise in non-communicable diseases (NCDs) like obesity, which can be understood as a profit-driven epidemic (Ibid). At present, there is little evidence available on the nature or extent of the impacts of commercial drivers on hospital food. Future research needs to comprehensively map and characterize hospital food environments and interrogate the links between the food provided and patient dietary health outcomes.

Appropriate funding should be provided to ensure a healthy, fair, sustainable, affordable, and culturally appropriate food environment for hospitalized children and their families. Beyond being a moral obligation for hospitals, this is likely to pay back in the long term through decreased hospitalizations, improved recovery rates, reduced social inequalities, and healthier children as well as adults. UNICEF [75] advocates for governments to develop and enforce policies and regulations that ensure nutritious and affordable food and healthy and sustainable food environments for all children. Hospital food environments could, and should, be one of the most important drivers of change in this realm. In the meantime, they should resist the excessive infiltration of the CDOH in public settings, protecting children and young people from business interests that go against population health [28].

## 5. Conclusions

Social and economic disconnection from the food system, the prioritization of business interests, and the infiltration of powerful economic operators in public institutions affect food environments and practices in healthcare contexts. The mechanisms through which the CDOH act as a barrier to healthy food and eating for children in hospitals in HICs are: via fostering obesogenic food environments; by displacing opportunities and sources of culturally inclusive food options; and by privileging individualized eating and convenience foods over commensal social eating. It is paramount that public hospitals are enabled to resist commercial pressures and norms in order to foster healthy and inclusive food environments. Otherwise, institutions that are designed to improve health will continue to be sites in which children and adolescents are exposed to the marketing and selling of foods that have been proven to be addictive and harmful, as well as the uncritical reproduction of individualized eating practices.

## Data Availability

Not applicable.
